# Predicting Factors of Functional Outcome in Patients with Acute Ischemic Stroke Admitted to Neuro-Intensive Care Unit—A Prospective Cohort Study

**DOI:** 10.3390/brainsci10120911

**Published:** 2020-11-26

**Authors:** Fabio Pilato, Serena Silva, Iacopo Valente, Marisa Distefano, Aldobrando Broccolini, Valerio Brunetti, Pietro Caliandro, Giacomo Della Marca, Riccardo Di Iorio, Giovanni Frisullo, Mauro Monforte, Roberta Morosetti, Carla Piano, Rosalinda Calandrelli, Fioravante Capone, Andrea Alexandre, Alessandro Pedicelli, Cesare Colosimo, Anselmo Caricato

**Affiliations:** 1 UOC Neurologia, Dipartimento Scienze dell’invecchiamento, Neurologiche, Ortopediche e della Testa-Collo, Fondazione Policlinico Universitario A. Gemelli IRCCS, 00168 Rome, Italy; marisa.distefano@hotmail.it (M.D.); aldobrando.broccolini@policlinicogemelli.it (A.B.); valerio.brunetti@policlinicogemelli.it (V.B.); pietro.caliandro@policlinicogemelli.it (P.C.); giacomo.dellamarca@policlinicogemelli.it (G.D.M.); riccardo.diiorio@policlinicogemelli.it (R.D.I.); giovanni.frisullo@policlinicogemelli.it (G.F.); mauro.monforte@policlinicogemelli.it (M.M.); roberta.morosetti@policlinicogemelli.it (R.M.); carla.piano@policlinicogemelli.it (C.P.); 2 Unit of Neurology, Neurophysiology, Department of Medicine, Università Campus Bio-Medico di Roma, 00128 Rome, Italy; f.capone@unicampus.it; 3 UOC Anestesia Rianimazione, Terapia Intensiva e Tossicologia Clinica, Dipartimento Scienze dell’Emergenza, Anestesiologiche e della Rianimazione, Fondazione Policlinico Universitario A. Gemelli IRCCS, 00168 Rome, Italy; serena.silva@policlinicogemelli.it (S.S.); Anselmo.caricato@policlinicogemelli.it (A.C.); 4 UOC Radiologia e Neuroradiologia, Dipartimento di Diagnostica per Immagini, Radioterapia Oncologica ed Ematologia, Fondazione Policlinico Universitario A. Gemelli IRCCS, 00168 Rome, Italy; Iacopo.valente@policlinicogemelli.it (I.V.); rosalinda.calandrelli@policlinicogemelli.it (R.C.); andrea.alexandre@policlinicogemelli.it (A.A.); alessandro.pedicelli@policlinicogemelli.it (A.P.); cesare.colosimo@policlinicogemelli.it (C.C.); 5 Università Cattolica del Sacro Cuore, Largo Francesco Vito, 1, 00168 Rome, Italy

**Keywords:** acute ischemic stroke, neuro-intensive care unit, thrombolysis, thrombectomy, outcome

## Abstract

Although thrombectomy is beneficial for most stroke patients with large vessel occlusion (LVO), it has added new issues in acute management due to intensive care support. In this prospective cohort study, we described the patients admitted to our neuro-intensive care unit (NICU) after thrombectomy in order to assess factors linked to functional outcomes. The outcome was independency assessed for stroke patients consecutively admitted to NICU for an ischemic stroke due to LVO of the anterior cerebral circulation that underwent intra-arterial mechanical thrombectomy (IAMT), either in combination with intravenous thrombolysis (IVT) in eligible patients or alone in patients with contraindications for IVT. Overall, 158 patients were enrolled. IVT (odds ratio (OR), 3.78; 95% confidence interval (CI), 1.20–11.90; *p* = 0.023) and early naso-gastric tube removal (OR, 3.32; 95% CI, 1.04–10.59 *p* = 0.042) were associated with good outcomes, whereas a high baseline National Institutes of Health Stroke Scale (NIHSS) score (OR, 0.72 for each point of increase; 95% CI, 0.61–0.85; *p* < 0.001) was a predictor of poor outcomes at 3 months. Older age (OR, 0.95 for each year of increase; 95% CI, 0.92–0.99; *p* = 0.020) and hemorrhagic transformation (OR, 0.31; 95% CI, 0.11–0.84; *p* = 0.022) were predictors of poor outcomes after IAMT, whereas a modified Treatment in Cerebral Infarction (mTICI) score of 2b/3 was a predictor of good outcomes (OR, 7.86; 95% CI, 1.65–37.39; *p* = 0.010) at 6 months. Our results show that acute stroke patients with LVO who require NICU management soon after IAMT may show specific clinical factors influencing short- and long-term neurologic independency.

## 1. Introduction

Stroke is a leading cause of death and long-term disability worldwide [1] and its social burden is huge in term of disability and mortality [2]. Over the last two decades, the management of stroke patients has shifted from secondary prevention to acute treatments. Intravenous thrombolysis (IVT) with recombinant tissue-type plasminogen activator (rt-PA) has been the only mainstay of treatment, initially within 3 h from symptom onset [3] and later extended to 4.5 h [4]. Recently, interventional trials implementing intra-arterial mechanical thrombectomy (IAMT) have made a significant step forward in acute stroke management, extending the therapeutic window beyond 4.5 h [5,6,7,8,9]. IAMT has become the standard of care for acute ischemic stroke (AIS) due to large vessel occlusion (LVO) in anterior circulation [6]. These effective time-dependent treatments, along with management in the setting of stroke-units, have consistently changed acute stroke treatment, improving the outcome of stroke patients in terms of survival and independency [10,11]. Current guidelines suggest that stroke patients who receive IVT undergo resource-intensive monitoring, including frequent vital signs checks and neurological examinations for early detection and management of potential complications in the first 24 h after IVT [12].

Admission of AIS patients treated with IVT or thrombectomy primarily to a neuro-intensive care unit (NICU) usually depends on local health care practices and the availability of resources [13], but it is estimated that 24% of all patients admitted with acute ischemic stroke may need critical care management [14].

Recent studies evaluated the effects of acute stroke patients features such as blood pressure [15], age, neurological status and vascular status assessed by neuroradiological techniques [5,7,8] as well as the type of acute treatments [16] on their outcomes. Other studies evaluated stroke risk factors [17] and clinical score [14] for early identification of patients at increased risk of developing intensive care unit (ICU) needs.

Although IAMT has been shown to be beneficial for most patients [18], it has added new issues since this acute management often requires intensive care support [13]. Indeed, recent studies evaluated the usefulness of intubation and general anesthesia (GA) or conscious sedation (CS) in spontaneously breathing patients during IAMT [19] and analyzed the effects of non-invasive versus invasive airway management during IAMT [20], without reaching an unequivocal conclusion [21]. On the other hand, in an ICU setting, co-morbidities such as hyperglycemia [22,23], hypertension [24], blood oxygenation [25], infections [26] and their management may influence short-term and long-term outcomes [27].

Overall, clinical factors associated with the outcomes of AIS patients requiring ICU treatment after IAMT are still poorly understood and studies in this population are lacking.

The aim of this study was to evaluate the profile of AIS patients admitted to an NICU after IAMT to define their features and the predictors of the neurological outcome.

## 2. Materials and Methods

This was a prospective, observational, single-center pilot study, conducted in the neurology and NICU departments at the local university high-volume tertiary stroke center between January 2017 and May 2019 with a follow-up period of 180 days.

The study population included all patients consecutively admitted to the NICU for an ischemic stroke due to LVO of the anterior cerebral circulation who underwent IAMT, either in combination with rt-PA in eligible patients and according to current guidelines, or alone in patients with contraindications for intravenous thrombolysis. Baseline clinical and neurological status, modified Rankin Scale (mRS) and National Institute of Health Stroke Scale Score (NIHSS) were registered at the time of arrival at hospital. A neuroimaging protocol including non-contrast-CT (NCCT) scan, CT angiography (CTA) and perfusion CT (PCT) were performed according to guidelines [11].

All patients treated by IAMT were evaluated for general anesthesia (GA) or sedation by an expert anesthesiologist. They received supplementary oxygen to achieve saturations above 94%, according to current guidelines [11]. Mechanical thrombectomy was performed with contact aspiration as a first-line strategy with a possible switching towards the combined technique in the case of inability to achieve successful recanalization. The modified Treatment in Cerebral Infarction (mTICI) score was assessed for all patients [28] and mTICI 2b/3 was considered a successful recanalization.

AIS patients enrolled in the study met the following criteria: (1) being at least 18 years old; (2) having AIS due to occlusion of a intracranial large vessel of anterior circulation confirmed by multimodal CT; (3) having a basal mRS score equal or less than 2; (4) having a National Institutes of Health Stroke Scale (NIHSS) score at admission of 5 or more; (5) having undergone endovascular treatment; (6) having been routinely admitted to NICU after an endovascular procedure.

Patients stayed in the NICU for at least 24 h, depending on their clinical condition, and then they were transferred to the intermediate stroke care unit. According to current guidelines [11], patients were daily evaluated for dysphagia. A nasogastric tube (NGT) was inserted in dysphagic patients and it was removed when the patient was able to swallow.

We evaluated the degree of functional recovery at 90 days (primary endpoint) and at 180 days (secondary endpoint), according to the mRS through a structured interview. Good recovery was defined as a mRS score ≤2. The relationship between clinical features and independency was also investigated. At the established follow-up period, all patients, relatives or caregivers were reached, and all interviews were completed.

Baseline demographics and clinical characteristics were compared between subjects with unfavorable (mRS score 3–6) and favorable (mRS score 0–2) outcomes at 90 and 180 days. Age, NIHSS, mechanical ventilation (days), length of stay in ICU (LOS-ICU), length of stay in hospital (LOS-H), hemoglobin at the entrance in emergency department (Hb-ECU) and hemoglobin at the entrance in intensive care unit (Hb-ICU) were all treated as continuous variables. All other variables were treated as binaries. mTICI score was categorized into mTICI 2b/3 and mTICI 0-2a.

For continuous measures, means and SD are presented and *p*-values calculated with a two-tailed *t*-test for Gaussian continuous variables and the Mann–Whitney U test for non-Gaussian continuous variables. Normality of distributions was assessed using histograms and the Shapiro–Wilk test. For categorical measures, frequencies and percentages are presented and *p*-values calculated with a χ^2^ or a two-tailed Fisher’s exact test as appropriate. No adjustments were made for multiple comparisons. The statistical significance threshold was set at *p* = 0.05.

A multivariate analysis with favorable outcome at 3 months or 6 months as dependent variables was performed. Other than age and sex, only variables with *p*-value less than 0.05 at univariate analysis were included in a forward stepwise logistic regression model. All variables (with the exception of confounding factors) included in the multivariate model with a variable-inflating factor (VIF) greater than 2.5 were excluded from the analysis due to multicollinearity issues. To improve the interpretability of the results, we measured the marginal effects of age and NIHSS in predicting good clinical outcome for both 90-day and 180-day time windows, keeping the other covariates fixed. Since we excluded patients with missing essential data from our analysis, we did not impute for missing data.

Statistical analysis was performed with STATA 15.1 (StataCorp LLC, College Station, TX, USA).

The study was approved by the hospital ethics committee (Fondazione Policlinico Universitario A. Gemelli—IRCCS, ID: 3004).

## 3. Results

**Figure 1:**
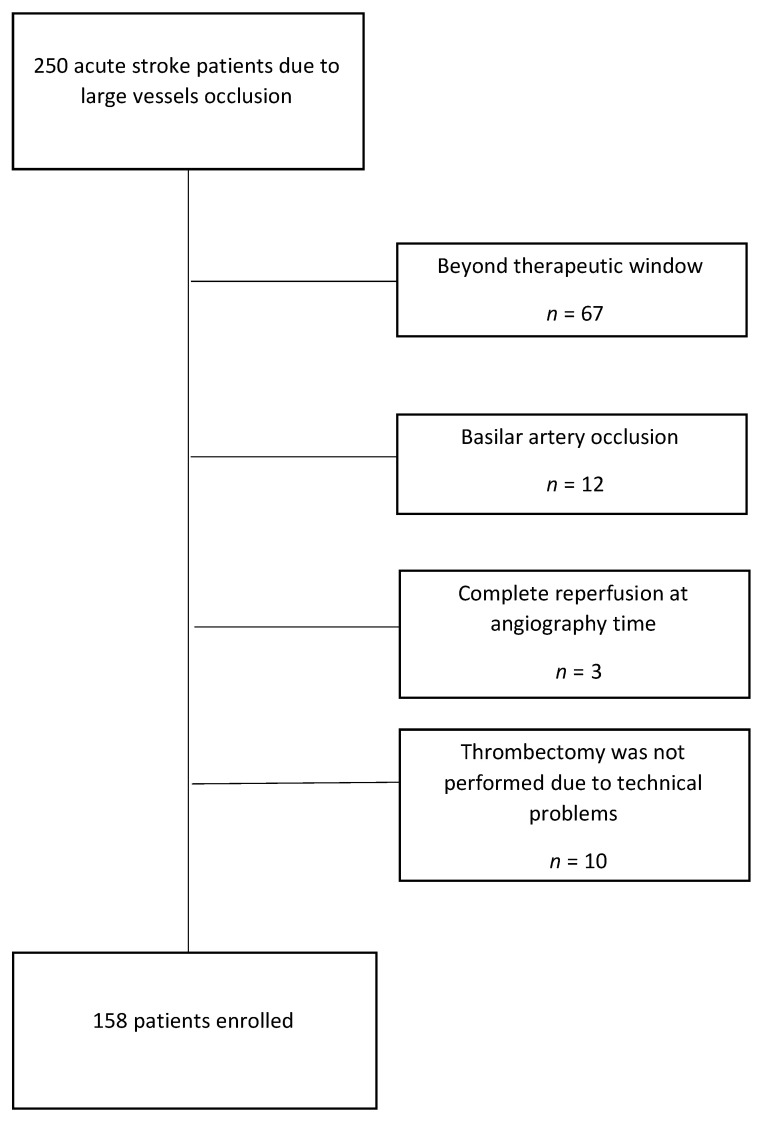
Flowchart of patients’ enrollment.

An enrollment diagram is presented in Figure 1. One hundred fifty-eight patients with a diagnosis of AIS fulfilled all inclusion criteria and were enrolled in the study.

The median age was 77 (Interquartile range (IQR) 66.5–84) and 86 patients (54.4%) were women. Twenty-three patients had a history of diabetes mellitus type 2 (14.6%), 16 patients (10.1%) had atrial fibrillation (AF) (either referred in their history or detected during hospitalization) and 63 patients (39.9%) showed hypertension. Endovascular recanalization with an mTICI 2b/3 score was achieved in 135 patients (85.4 %) (Table 1).

### 3.1. Predictors of Independency at 3 Months

**Figure 2:**
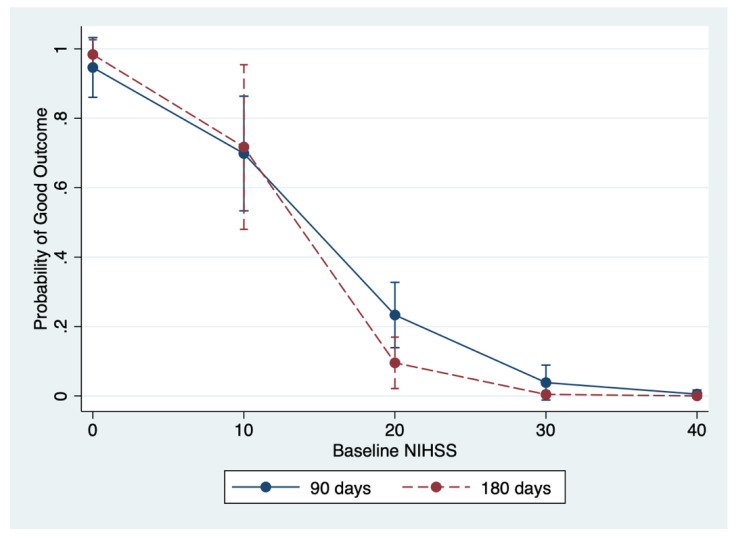
Marginal effect of baseline NIHSS on the probability of a good outcome at 90 days (blue line) and 180 days (red line); the other covariates of the multivariable model are kept fixed. A good outcome is defined as eventual return to home or discharge to assisted living (modified Rankin scale (mRS) < 2).

The primary outcome was independency at 3 months, recorded as mRS < 2. Univariate analysis revealed differences between poor and good outcome groups at 3 months in the following features: age (*p* < 0.001), IVT (*p* = 0.046), NGT removal (*p* =0.001), baseline NIHSS score (*p* < 0.001) and mTICI 2b/3 score (*p* = 0.001) (Table 1). All these factors, along with sex, were entered into a multivariate logistic regression analysis (with the exception of the length of mechanical ventilation due to multicollinearity) and the results showed that IVT (OR, 2.87; 95% CI, 1.05–7.83; *p* = 0.039) and NGT-removal (OR, 2.96; 95% CI, 1.07–8.13; *p* = 0.035) were associated with good outcomes, whereas a high baseline NIHSS score (OR, 0.80 for each point of increase; 95% CI, 0.72–0.90; *p* < 0.001) was a predictor of poor outcomes after IAMT (Table 2). According to these results, we developed a predictive model based on the incremental baseline NIHSS score (Figure 2), considering the other covariates of the multivariate logistic models at their mean value.

### 3.2. Predictors of Independency at 6 Months

A secondary outcome was independency at 6 months, recorded as mRS < 2 (Table 3). Univariate analysis revealed differences between groups with poor and good outcomes at 6 months: age (*p* < 0.001), diabetes (*p* = 0.012), hemorrhagic transformation (*p* = 0.028), NGT removal (*p* = 0.005), baseline NIHSS score (*p* < 0.001) and mTICI 2b/3 (*p* = 0.001) (Table 3).

**Figure 3:**
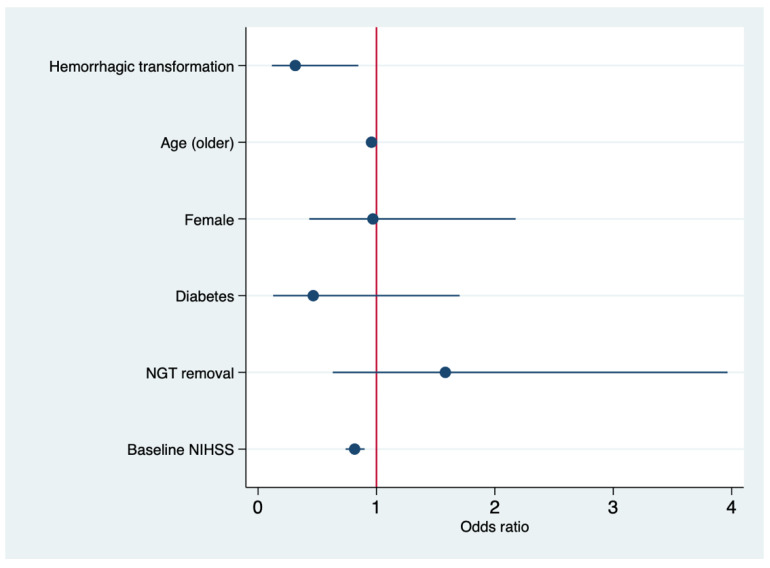
Forest plot showing odds ratios of all clinical variable included in the predictive model for a 180-day good outcome.

All these factors, along with sex, were entered into a multivariate logistic regression analysis and the results showed that older age (OR, 0.95 for each year of increase; 95% CI, 0.92–0.99; *p* = 0.020), hemorrhagic transformation (OR, 0.31; 95% CI, 0.11–0.84; *p* = 0.022), increased baseline NIHSS score (OR, 0.81 for each point of increase; 95% CI, 0.74–0.90; *p* < 0.001) were predictors of poor outcomes after IAMT, whereas a mTICI score of 2b/3 was a predictor of good outcomes (OR, 7.86; 95% CI, 1.65–37.39; *p* = 0.010) (Table 4 and Figure 3).

**Figure 4:**
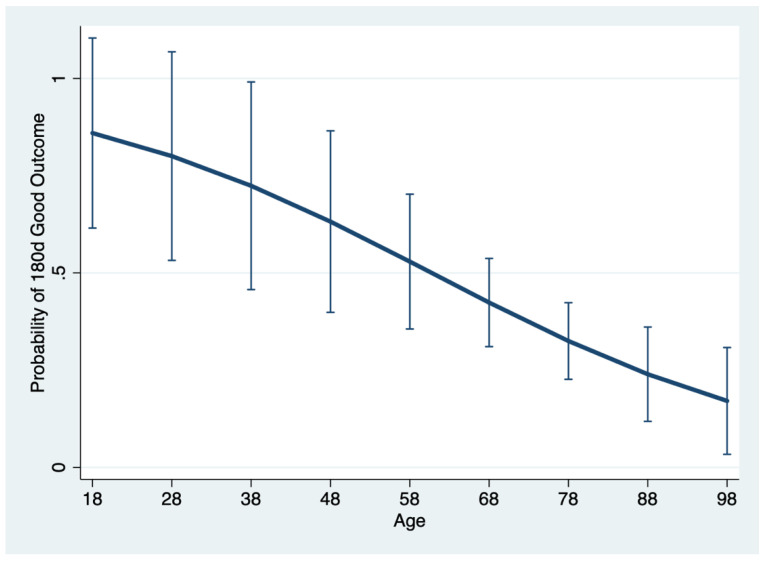
Marginal effect of age on the probability of a good outcome at 180 days; the other covariates of the multivariable model are kept fixed. A good outcome is defined as eventual return to home or discharge to assisted living (mRS < 2).

Figure 2 outlines the probability of 90-day and 180-day good outcomes as a function of incremental admission NIHSS score and Figure 4 outlines the probability of a good outcome at 180days as a function of the patient’s age, both considering the other covariates of the multivariate logistic models at their mean value.

## 4. Discussion

In our study, we have evaluated clinical factors of acute stroke patients admitted to the NICU after IAMT to find the determinants for 90-day and 180-day functional outcomes.

At the 90-day evaluation, IV thrombolysis and NGT removal were associated with good functional outcomes, whereas a high baseline NIHSS score was associated with poor functional outcomes. At the 180-day evaluation, reperfusion was associated with good functional outcomes, whereas older age, hemorrhagic transformation and a high baseline NIHSS score were associated with poor functional outcomes.

Interestingly, taking into account patients with poor outcomes at both 90-day and 180-day evaluations, about 80% of these patients showed nearly complete reperfusion, having a mTICI score 2b or 3. Therefore, in the setting of the NICU, reperfusion seems to be not the only determinant of good outcomes. Indeed, some patients’ intrinsic characteristics, as well as some aspects of NICU management, may have a role that can counterbalance the positive effects of reperfusion and have a different impact on short- and long-term outcomes.

Dysphagia is a common complication after acute stroke, as it can affect more than 50% of patients and is related to an increased risk of stroke-associated pneumonia (SAP) [29]. The relationship between NGT placement and the risk of SAP is controversial [30,31]. Moreover, it is unclear what factors contribute to pneumonia or which measures may reduce its frequency [32,33]. We found that NGT removal was associated with better functional outcomes at 90 days, but length of stay in hospital and long-term outcomes were comparable between groups, showing that NGT removal does not affect the hospitalization but rather the time needed to regain independency in the short-term period. This finding may have several explanations, but it is conceivably related to the risk reduction of developing pneumonia [34] in the post-stroke period that reduces patients’ activities and delays recovery.

Reperfusion was the main factor influencing a 180-day good outcome, whereas older age, hemorrhagic transformation and incremental NIHSS score were linked with 180-day poor outcomes. Timely artery recanalization of LVO improves functional outcomes and reduces mortality [5,6,7,8,9]. However, a recent study has been reported that, after IAMT, 21.7% of patients developed large MCA infarcts needing decompressive hemicraniectomy (DHC) [35]. Interestingly, 15.8% of these patients showed successful reperfusion after IAMT, demonstrating that a large infarct may develop despite achieving successful recanalization, independently from the time from stroke onset to groin puncture [35]. These observations reveal that although reperfusion plays a significant role, other clinical factors are important for a good outcome too.

Another important point is that different and distinctive factors may influence either short-term or long-term outcomes or both. Our study indicates that baseline NIHSS score has a significant and incremental effect on independency both in the short- and long-term and high baseline NIHSS scores reduce long-term chances of independency, particularly in elderly patients.

Older age was a predictor of poor outcomes in the long-term, but its effect was not evident in the short-term. This may be due to a cumulative effect of age, because its impact is low in the short-term and increases over time. On the other hand, a high baseline NIHSS score influences both short- and long-term outcomes.

The evidence of hemorrhagic transformation (HT) of the ischemic lesion was significantly associated with poor outcomes at 180 days. This finding deserves a particular remark, because neurologic worsening due to intracranial hemorrhage after AIS is associated with poor outcomes and mortality rates of up to 50%, especially in patients with parenchymal hematoma type 2 [36], which represents the majority of all symptomatic intracranial hemorrhages [37].

It is still unclear how to identify patients who will develop HT and several causes have been proposed, such as genetics [38], baseline ischemic core [39], age and stroke severity [40], blood pressure [41,42], IVT [43] and inflammation [44].

Regarding IVT, a previous study reported no significant differences in HT when IAMT was preceded by IVT and, in a selected population without pre-stroke anticoagulation, a better 90-day functional outcome was reported [43]. Our data are in agreement with these results.

Conversely, it has been reported that previous treatment with aspirin monotherapy increases the bleeding risk of rt-PA in both observational and randomized trials with no effect on clinical outcome and the risk of intracerebral hemorrhage is increased with the combination of aspirin and clopidogrel [45]. Moreover, in an experimental model of stroke, Zheng et al. demonstrated that mice pretreated with dual antiplatelet therapy showed an increased risk of hemorrhagic transformation when treated with tPA [46]. These results may suggest a cautious use of anticoagulant drugs in the acute stage, avoiding the unnecessary combination of antithrombotic treatments. In addition, timely correction of coagulopathy should be considered, especially in patients who undergo IVT or with active anticoagulant use for either deep venous thrombosis prevention or atrial fibrillation.

Despite promising strides forward in IAMT implementation in clinical practice, we still have many unanswered questions in the management of patients with acute ischemic stroke, and also for those patients in whom recanalization is reached.

This study has some limitations—firstly, the observational design and the consequent use of post-hoc hypotheses; secondly, it was a single-center study and the sample size was relatively small. On the other hand, this design allowed us to complete follow-up on the majority of our patient population, even if results might have been influenced by premature deaths of some patients during follow-up, and further studies are warranted to confirm these observations.

## 5. Conclusions

Our results show that acute stroke patients with LVO who require NICU management soon after IAMT may have specific clinical factors influencing short- and long-term neurologic independency. Future studies should address whether tailored therapeutic approaches may affect these factors, in order to further improve neurological outcomes.

## Figures and Tables

**Table 1:** Baseline, procedural and outcome parameters at 90-day follow-up.

Characteristics	Overall	Poor Outcomes at 90 Days	Good Outcomes at 90 Days	*p*-Value
***N***	158	107 (67.72%)	51 (32.28%)	
**Age**	73.80 ± 12.60	76.33 ± 11.52	68.42 ± 13.19	<0.001
**Females**	86 (54.43%)	59/107 (55.14%)	27/51 (52.94%)	0.795
**Diabetes**	23 (14.56%)	20/107 (18.69%)	3/51 (5.88%)	0.051
**Hypertension**	63 (39.87%)	43/107 (40.19%)	20/51 (39.22%)	10.000
**Smoking**	16 (12.21%)	11/107 (12.36%)	5/51 (11.90%)	0.941
**Dyslipidemia**	22 (13.92%)	17/107 (15.89%)	5/51 (9.80%)	0.302
**AF**	16 (10.13%)	10/107 (9.35%)	6/51 (11.76%)	0.637
**Seizures**	2 (1.27%)	2/107 (1.87%)	0	-
**AED**	1 (0.63%)	0	1/51 (1.96%)	-
**NGT at discharge**	11 (6.96%)	9/107 (8.41%)	2/51 (3.92%)	0.505
**NGT removal**	42 (26.58%)	20/107 (18.69%)	22/51 (43.14%)	0.001
**Tracheostomy**	15 (9.55%)	13/106 (12.26%)	2/51 (3.92%)	0.146
**Intubation**	117 (74.05%)	84/107 (78.50%)	33/51 (64.71%)	0.064
**IV Thrombolysis**	81 (51.27%)	49/107 (45.79%)	32/51 (62.75%)	0.046
**DHC**	5 (3.16%)	3/107 (2.80%)	2/51 (3.92%)	0.658
**Pneumonia**	17 (10.90%)	15/106 (14.15%)	2/50 (4.00%)	0.095
**Sepsis**	1 (0.63%)	1/107 (0.93%)	0	-
**Septic Shock**	-	-	-	-
**Hemorrhagic transformation**	41 (26.11%)	30/106 (28.30%)	11/51 (21.57%)	0.368
**HT1-PH1**	16 (10.13%)	10/107 (9.35%)	6/51 (11.76%)	0.779
**PH2**	11 (6.96%)	9/107 (8.41%)	2/51 (3.92%)	0.505
**24 h vasopressors infusion**	13 (8.23%)	11/107 (10.28%)	2/51 (3.92%)	0.226
**NIHSS**	16.74 ± 5.58	18.48 ± 4.72	13.07 ± 11.52	<0.001
**Mortality**	40 (25.32%)	40 (100%)	-	-
**mTICI 2b/3**	135 (85.44%)	84/107 (78.50%)	50/51 (98.04%)	0.001
**Site of occlusion**				0.090
**MCA**	111 (70.25%)	72/107 (67.29%)	39/51 (76.47%)	
**ICA**	47 (29.75%)	35/107 (32.71%)	12/51 (23.53%)	
**Left-side occlusion**	88 (55.70%)	63/107 (58.88%)	25/51 (49.02%)	0.243
**Mechanical ventilation (n. pts)**	92 (58.23%)	64/107 (59.81%)	28/51 (54.90%)	0.607
**Mechanical ventilation (days)**	2.05 ± 3.84	2.28 ± 3.64	1.48 ± 4.31	0.039
**LOS-ICU**	3.35 ± 4.74	3.69 ± 4.64	2.56 ± 4.93	<0.001
**LOS-H**	13.31 ± 13.89	13.75 ± 13.56	12.27 ± 14.75	0.39
**Hb-ECU**	13.23 ± 1.66	13.24 ± 2.11	13.21 ± 1.66	0.551
**Hb-ICU**	12.33 ± 1.85	12.31 ± 1.87	12.38 ± 1.82	0.961
**Trop+ (>0.00)**	60 (37.97%)	46/107 (42.99%)	14/51 (27.45%)	0.060

AF: atrial fibrillation; AED: antiepileptic drugs; mTICI: modified Treatment in Cerebral Infarction; MCA: middle cerebral artery; ICA: internal carotid artery; NGT: nasogastric tube; NIHSS, National Institutes of Health Stroke Scale; DHC, decompressive hemicraniectomy; LOS-ICU, length of stay in ICU; LOS-H, length of stay in hospital; Hb-ECU: hemoglobin at hospital arrival; Hb-ICU: hemoglobin at ICU arrival; Trop+: HS-troponin. For continuous variables, *p*-values were calculated with a two-tailed *t*-test for Gaussian continuous variables and with a Mann–Whitney U test for non-Gaussian continuous variables; for categorial variables, *p*-values were calculated with a χ^2^ or a two-tailed Fisher’s exact test, as appropriate.

**Table 2:** Multivariate logistic regression model for predictors of 90-day good outcome (mRS 0–2).

Var.	OR	95% CI	*p*-Value
**Age (older)**	0.97	0.92–1.02	0.247
**Female**	0.69	0.23–2.03	0.510
**IV Thrombolysis**	3.78	1.20–11.90	0.023
**NGT-removal**	3.32	1.04–10.59	0.042
**LOS-ICU**	0.90	0.69–1.18	0.467
**mTICI 2b/3**	5.87	0.63–54.54	0.120
**Mechanical ventilation**	1.02	0.74–1.42	0.866
**Baseline NIHSS**	0.72	0.61–.85	<0.001

LOS-ICU, length of stay-ICU; mTICI: modified Treatment in Cerebral Infarction; OR, odds ratio; CI, confidence interval; NIHSS, National Institutes of Health Stroke Scale; NGT: nasogastric tube.

**Table 3:** Baseline, procedural and outcome parameters at 180-day follow-up.

Characteristics	Poor Outcome at 180 Days	Good Outcome at 180 Days	*p*-Value
**N**	**93 (58.86%)**	**65 (41.14%)**	
**Age**	76.92 ± 11.46	69.19 ± 12.87	<0.001
**Females**	51/93 (54.84%)	35/65 (53.85%)	0.902
**Diabetes**	19/93 (20.43%)	4/65 (6.15%)	0.012
**Hypertension**	40/93 (43.01%)	23/65 (35.38%)	0.335
**Dyslipidemia**	17/93 (18.28%)	5/65 (7.69%)	0.065
**AF**	11/93 (11.83%)	5/65 (7.69%)	0.437
**AED**	0	1 (1.54%)	-
**Seizures**	0	2/65 (3.08%)	-
**NGT at discharge**	9/93 (9.68%)	2/65 (3.08%)	0.126
**NGT removal**	17/93 (18.28%)	25/65 (38.46%)	0.005
**Tracheostomy**	8/92 (8.70%)	7/65 (10.77%)	0.663
**Intubation**	74/93 (79.57%)	43/65 (66.15%)	0.058
**IV Thrombolysis**	44/93 (47.31%)	37/65 (56.92%)	0.234
**DHC**	2/93 (2.15%)	3/65 (4.62%)	0.403
**Pneumonia**	13/92 (14.13%)	4/64 (6.25%)	0.190
**Sepsis**	1/93 (1.08%)	0	-
**Septic Shock**	0	0	-
**HT**	30/92 (32.61%)	11/65 (16.92%)	0.028
**HT1-PH1**	10/92 (10.75%)	6/65 (9.23%)	0.755
**PH2**	9/92 (9.68%)	2/65 (3.08%)	0.109
**24h vasopressors infusion**	8/93 (8.60%)	5/65 (7.69%)	10.000
**NIHSS**	18.83 ± 4.74	13.73 ± 5.35	<0.001
**mTICI 2b/3**	72/93 (77.42%)	62/65 (95.38%)	0.001
**Site of occlusion**			0.238
**MCA**	62/93 (66.67%))	16/65 (24.62%)	
**ICA**	31/93 (33.33%)	16/65 (24.62%)	
**Left-side occlusion**	54/93 (58.06%)	34/65 (52.31%)	0.473
**Mechanical ventilation (n. pts)**	57/93 (61.29%)	35/65 (53.85%)	0.350
**Mechanical ventilation (days)**	1.87 ± 2.69	2.37 ± 5.29	0.256
**LOS-ICU**	3.13 ± 3.44	3.48 ± 6.03	0.056
**LOS-H**	13.32 ± 14.33	13.29 ± 13.29	0.258
**Hb-ECU**	13.11 ± 2.15	13.41 ± 1.68	0.598
**Hb-ICU**	12.20 ± 1.93	12.52 ± 1.72	0.355
**Trop + (>0.00)**	37/93 (39.78%)	23/65 (35.38%)	0.575

AF: atrial fibrillation; AED: antiepileptic drugs; mTICI: modified Treatment in Cerebral Infarction; MCA: middle cerebral artery; ICA: internal carotid artery; NGT: nasogastric tube; NIHSS, National Institutes of Health Stroke Scale; DHC, decompressive hemicraniectomy; LOS-ICU, length of stay in ICU; LOS-H, length of stay in hospital; Hb-ECU: hemoglobin at hospital arrival; Hb-ICU: hemoglobin at ICU arrival; Trop+: HS-troponin; HT: hemorrhagic transformation. For continuous variables, *p*-values were calculated with a two-tailed *t*-test for Gaussian continuous variables and with a Mann–Whitney U test for non-Gaussian continuous variables; for categorial variables, *p*-values were calculated with a χ^2^ or a two-tailed Fisher’s exact test, as appropriate.

**Table 4:** Multivariable logistic regression for predictors of 180-day good outcome (mRS 0–2).

var.	OR	95% CI	*p*-Value
**Age (older)**	0.95	0.92–0.99	**0.020**
**Female**	0.97	0.43–2.17	0.965
**Diabetes**	0.46	0.12–1.75	0.248
**Hemorrhagic transformation**	0.31	0.11–0.84	**0.022**
**NGT-removal**	1.58	0.63–3.96	0.328
**mTICI2b/3**	7.86	1.65–37.39	**0.010**
**Baseline NIHSS**	0.81	0.74–0.90	**<0.001**

mTICI: modified Treatment in Cerebral Infarction; OR, odds ratio; CI, confidence interval; NIHSS, National Institutes of Health Stroke Scale; NGT: nasogastric tube.
